# Effects of Pollen Sources on Fruit Set and Fruit Characteristics of ‘Fengtangli’ Plum (*Prunus salicina* Lindl.) Based on Microscopic and Transcriptomic Analysis

**DOI:** 10.3390/ijms232112959

**Published:** 2022-10-26

**Authors:** Lijun Deng, Tie Wang, Juan Hu, Xinxia Yang, Yuan Yao, Zhenghua Jin, Zehao Huang, Guochao Sun, Bo Xiong, Ling Liao, Zhihui Wang

**Affiliations:** College of Horticulture, Sichuan Agricultural University, Chengdu 611130, China

**Keywords:** ‘Fengtangli’ plum, fruit set, fruit quality, metaxenia, pollination, pollen tube growth, transcriptomic analysis

## Abstract

Adequate yield and fruit quality are required in commercial plum production. The pollen source has been shown to influence fruit set and fruit characteristics. In this study, ‘Siyueli’, ‘Fenghuangli’ and ‘Yinhongli’ were used as pollinizers of ‘Fengtangli’ plum. Additionally, self-pollination, mixed pollination, and open pollination were performed. We characterized the differences in pollen tube growth, fruit set and fruit quality among pollination combinations. ‘Fengtangli’ flowers pollinated by ‘Fenghuangli’ had more pistils with pollen tubes penetrating the ovary and the highest fruit set rate, while the lowest fruit set rate was obtained from self-pollination. In self-pollinated flowers, 33% of pistils had at least one pollen tube reaching the ovary, implying that ‘Fengtangli’ is partially self-compatible. Pollen sources affected ‘Fengtangli’ fruit size, weight, pulp thickness, soluble solids, and sugar content. Transcriptome analysis of ‘Siyueli’-pollinated and ‘Yinhongli’-pollinated fruits revealed 2762 and 1018 differentially expressed genes (DEGs) involved in the response to different pollen sources. DEGs were enriched in plant hormone signal transduction, starch and sucrose metabolism, and MAPK signaling pathways. Our findings provide a reference for the selection of suitable pollinizers for ‘Fengtangli’ plum and promote future research on the metaxenia effect at the molecular level.

## 1. Introduction

Japanese plum (*Prunus salicina* L.), native to China, is one of the most economically important stone fruits in the Rosaceae family worldwide [1]. Plums, one of the traditional fruits, have been grown in China for at least 3000 years. China ranks first among Japanese plum-producing countries, with a harvested area of 1.94 million ha and a production of 6.5 million tons in 2020 [2]. Plum fruit is rich in biologically active substances, such as vitamin C, carotenoids, polyphenols, and dietary fiber [3,4,5]. With high antioxidant activity, plums have the potential to promote human health by preventing cancer, diabetes, hypertension, and inflammation [6,7,8]. Plums are mostly consumed fresh, but they can also be processed into commercial products, such as canned fruit, juice, and fruit wine.

‘Fengtangli’ is a superb Japanese plum cultivar from Guizhou Province, China. The ‘Fengtangli’ plum is highly appreciated in China due to its exceptional fruit qualities, such as being large, crispy, juicy, and sweet. Field observations suggested that fruit set of ‘Fengtangli’ with different pollinizers varied significantly in many orchards, as well as fruit taste. Given that the result was disturbed by many uncontrollable factors, we explored the topic through pollination experiments in the present study.

An adequate fruit set depends on successful pollination and fertilization. Most Japanese plum cultivars exhibit typical gametophytic self-incompatibility (GSI), which prevents inbreeding and promotes outcrossing to generate genetic diversity [9,10]. The lack of cross-pollination may result in a poor fruit set. However, several stone fruit cultivars have shown self-compatibility in previous studies, such as ‘Methley’ plum [11], ‘Cristobalina’ sweet cherry [12], ‘Ceglédi arany’ apricot [13], and ‘Belona’ almond [14]. Self-incompatible plants obtain commercial yields by cross-pollination, and some self-compatible plants can also increase fruit set when they are cross-pollinated [15,16]. For high yields, it is critical to grow Japanese plum and its compatible pollinizers in the same orchard [17].

Several methods have been developed to identify compatible and suitable pollinizers. In the field, (in)compatibility can be evaluated preliminarily by recording the fruit set rate of crosses [18,19]. The final fruit set is usually attained 5 weeks after pollination [20]. In addition, compatible pollen sources can be accurately distinguished by examining pollen tube growth in the style under a fluorescence microscope. In gametophytic self-incompatible cultivars and incompatible crosses, pollen tube growth is prevented in the style and no pollen tubes reach the base of the style. Pollen tubes, on the other hand, can penetrate the ovary in self-compatible cultivars and compatible crosses [21]. As a result, observation of pollen tube behavior has proven to be a reliable indicator for determining the (in)compatibility of pollination combinations [22].

Pollen sources can affect fruit set rates. This was observed in ‘Zuili’ plums [23]. The fruit set rate of ‘Zuili’ pollinated with ‘Black Amber’ was 14.6%, while it was only 8.8% when pollinated with ‘Hongxinli’. According to Mira et al. [24], pollen sources also influenced pollen tube growth rate in sweet cherry. It is well known that pollen sources may have a direct influence on the fruit and seed features of the maternal plant, namely xenia [25]. Xenia includes metaxenia, which is the effect of pollen on fruit characteristics, such as size, shape, color, and sugar content [26,27]. The use of metaxenia effect in production is an effective means of improving fruit quality. Previous studies on metaxenia have been extensively documented in many species, including citrus [28], apple [29], grape [30], pear [31], and blueberry [32]. Kodad et al. [33] demonstrated that pollen sources had a clear effect on the fatty acid composition of almond kernels. In *Prunus salicina* L., however, little is known about metaxenia. Therefore, it is worthwhile to investigate the metaxenia effect and select appropriate pollen sources.

Although the molecular mechanism of the metaxenia effect has not been thoroughly studied, some scholars have proposed two hypotheses to explain this phenomenon. Denny synthesized previous studies and suggested that the difference in endogenous hormone levels in fruits leads to metaxenia effect, such as auxin, cytokinin, and gibberellin. Larger fruits have higher levels of hormones [25]. Previous studies have shown that RNA can be transported from cell to cell via intercellular plasmodesma and phloem for long-distance delivery and regulation of gene expression [34]. Liu [35] hypothesized that the signals that trigger the metaxenia effect may be mRNAs. These mRNAs could be released by the pollen tube and transported to the seeds and other parent tissues. The translocated mRNAs are functional, which can cause changes in fruit or seed characteristics [36].

Pollen source plays a crucial role in plum yield and fruit quality. However, there are few studies on this aspect. Based on field observations, we found that ‘Siyueli’, ‘Fenghuangli’, and ‘Yinhongli’ are commonly used as pollinizers for ‘Fengtangli’ in orchards. Therefore, these three cultivars were selected as pollen donors to pollinate ‘Fengtangli’ flowers. Additionally, self-pollination, mixed pollination and open pollination treatments were conducted. The objective of this study was to compare the differences in fruit set and fruit characteristics among various pollination combinations. Moreover, we observed pollen tube growth in the style and ovary of ‘Fengtangli’ flowers to identify probable self and cross (in)compatibility among the cultivars. In addition, by transcriptome sequencing, we identified significant differences in fruit transcript levels in plant hormone signal transduction, starch and sucrose metabolism pathways caused by metaxenia effect. The findings can be used to select suitable pollinizers for commercial production of ‘Fengtangli’ plum and to promote further research on the metaxenia effect at the molecular level.

## 2. Results

### 2.1. Pollen Germinability

**Figure 1:**
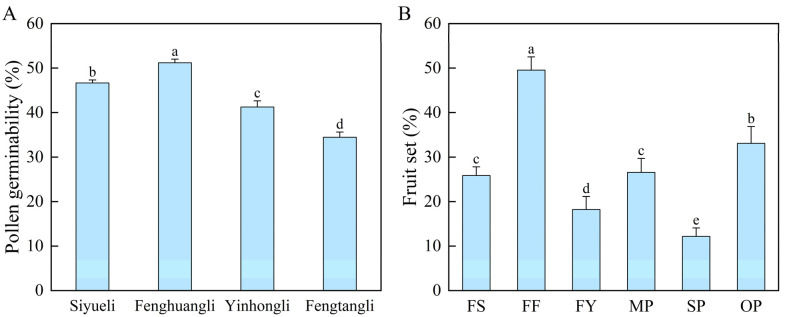
(**A**) Pollen germinability of four plum cultivars. (**B**) Fruit set rates of ‘Fengtangli’ plum with six pollination treatments. FS: ‘Fengtangli’ × ‘Siyueli’; FF: ‘Fengtangli’ × ‘Fenghuangli’; FY: ‘Fengtangli’ × ‘Yinhongli’; MP: mixed pollination; SP: self-pollination; OP: open pollination. Different letters above the bars indicate significant differences between treatments, *p* < 0.05 (the same below).

In pollen germination experiment in vitro, pollen germinability varied significantly among cultivars (Figure 1A). The pollen germinability of ‘Fenghuangli’ plum was the highest (51.22%), followed by ‘Siyueli’ (46.68%). ‘Yinhongli’ plum (41.27%) and ‘Fengtangli’ plum had the lowest pollen germinability (34.47%).

### 2.2. Fruit Set

The fruit set of ‘Fengtangli’ was significantly affected by pollen sources (Figure 1B). Flowers pollinated by ‘Fenghuangli’ had the highest fruit set rate (49.56%), followed by open pollination (33.10%). Moderate levels of fruit set were obtained in ‘Siyueli’ (25.87%) and mixed pollen (26.58%), with no significant differences. The ‘Fengtangli’ × ‘Yinhongli’ combination had a lower fruit set rate (18.22%), whereas the lowest was recorded in self-pollination (12.20%).

### 2.3. Pollen Tube Growth In Vivo

**Figure 2:**
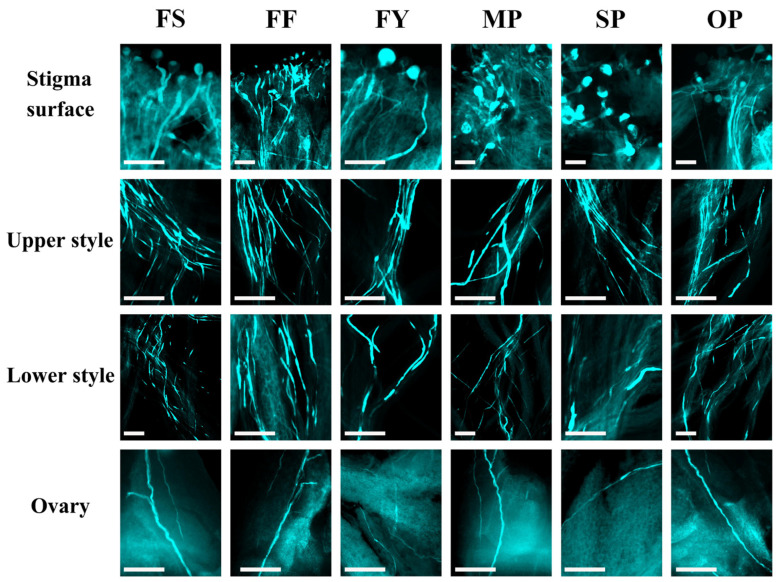
Fluorescence microscopic images of pollen germination and pollen tube growth of ‘Fengtangli’ plum with various pollination treatments. Scale bars, 100 µm.

To evaluate the compatibility of crosses, pollen tube growth was observed under a fluorescence microscope (Figure 2). The number of pollen tubes decreased gradually from the stigma to the base of the style and differed markedly among the six combinations (Table 1). More than 90% of pistils with pollen tubes at the upper third of the style were observed in ‘Fengtangli’ flowers when pollinated with ‘Siyueli’, ‘Fenghuangli’, ‘Yinhongli’, and mixed pollen, followed by open pollination (83.33%) and self-pollination (79.17%). The highest percentage of pistils with pollen tubes at the base of the style were found in ‘Fengtangli’ × ‘Fenghuangli’ combination (78%) and mixed pollination (76%), and the same two crosses also had the most pistils with pollen tubes penetrating the ovary (43% and 44%, respectively). Self-pollination had the fewest pistils with at least one pollen tube at the base of the style (33%) and ovary (18%).

Moreover, pollen tubes from ‘Fenghuangli’ pollen penetrated the upper third (47.33) and base (23.17) of the style more successfully, as well as the ovary (2.57). The ‘Yinhongli’- and self-pollinated flowers, however, showed a low number of pollen tubes in different parts of the style. Notably, the number of self-pollinated pollen tubes decreased sharply from the upper third to the base of the style, and irregular callose accumulation was observed at the tip of most pollen tubes, resulting in pollen tube growth cessation.

### 2.4. Metaxenia Effect on Fruit Characteristics

**Figure 3:**
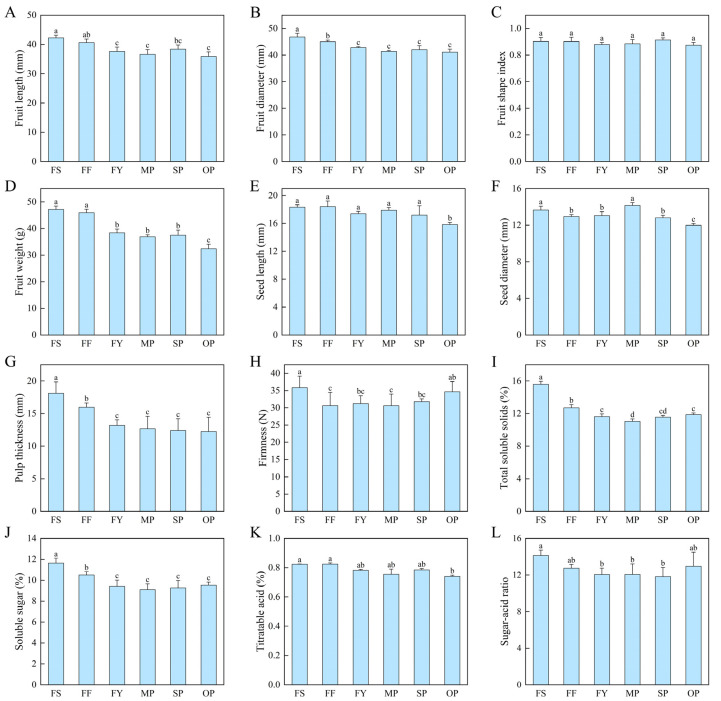
Fruit length (**A**), fruit diameter (**B**), fruit shape index (**C**), weight (**D**), seed length (**E**), seed diameter (**F**), pulp thickness (**G**), firmness (**H**), total soluble solids content (**I**), soluble sugar content (**J**), titratable acid content (**K**), and sugar-acid ratio (**L**) of ‘Fengtangli’ plum with different pollination treatments. Error bars indicate the standard deviation of total substance content, and different letters indicate significant differences in fruit characteristics, *p* < 0.05.

Pollen sources influenced fruit quality. The average length and diameter of ‘Fengtangli’ fruits ranged from 35.92 to 42.25 mm and 41.11 to 46.80 mm, respectively (Figure 3A,B). However, no discernible variations in fruit shape index were discovered between pollination combinations (Figure 3C). Fruits resulting from ‘Siyueli’ and ‘Fenghuangli’ pollination were significantly larger and heavier than other combinations (Figure 3D). Open pollination had the lowest single fruit weight (32.40 g), accounting for 68.72% of ‘Siyueli’ pollinated fruits, as well as the smallest seed size (Figure 3E,F). The pulp thickness of ‘Siyueli’ pollinated fruits was greatest (18.11 mm), followed by ‘Fenghuangli’ (15.98 mm) (Figure 3G). In addition, ‘Siyueli’ pollination resulted in the firmest fruit (35.84 N), whereas mixed pollination produced the least firm fruit (30.61 N) (Figure 3H).

Total soluble solids content (TSS) of fruit produced by mixed and self-pollination was significantly lower than in other treatments, at 11.03% and 11.57%, respectively. Fruits from the ‘Fengtangli’ × ‘Siyueli’ combination had the highest TSS content, 15.60%, which was 1.41 times higher than that of mixed pollination (Figure 3I). The soluble sugar content showed the same pattern. The soluble sugar content of ‘Siyueli’ (11.63%) and ‘Fenghuangli’ (10.51%) pollinated fruits was significantly higher than others (Figure 3J). Pollen sources did not affect titratable acid content (TA), and no significant difference was determined in the TA of ‘Fengtangli’ fruit from all combinations (Figure 3K). Overall, fruits pollinated by ‘Siyueli’ had the highest sugar-acid ratio (14.7) (Figure 3L).

### 2.5. Correlation Analysis

**Figure 4:**
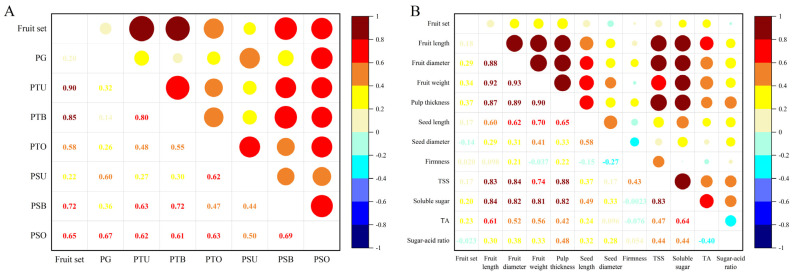
(**A**) Pearson’s correlation matrix between fruit set and reproductive parameters. PTU: number of pollen tubes at the upper third of style; PTB: number of pollen tubes at the base of style; PTO: number of pollen tubes penetrated to the ovary. PSU: percentage of pistils with pollen tubes at the upper third of style; PSB: percentage of pistils with pollen tubes at the base of style; PSO: the percentage of pistils with at least one pollen tube in the ovary. The values are statistically significant at *p* ≤ 0.05. (**B**) Pearson’s correlation matrix between fruit set and fruit characteristics.

High positive significant correlations were determined between fruit set and all the reproductive parameters, except the percentage of pistils with pollen tubes at the upper style (Figure 4A). Furthermore, there were positive significant correlations between the number of pollen tubes in the upper third of the style and the number of pollen tubes at the base of the style and in the ovary. The percentage of pistils with at least one pollen tube penetrating the ovary was highly correlated with other parameters.

There was no statistically significant correlation between fruit set and fruit characteristics (Figure 4B). In terms of fruit quality, fruit length and diameter, pulp thickness, seed length, and fruit weight all displayed a positive significant correlation with each other. Positive significant correlations were found between these quantitative traits and soluble solids as well as soluble sugar. However, no correlation was discovered between firmness and other traits.

### 2.6. Effects of Metaxenia on Transcriptomic Alterations

In order to explore the potential mechanism of the metaxenia effect, transcriptome analysis was performed on ‘Fengtangli’ plum. High-quality total RNA was isolated from the FS and FY fruits at S5 (harvested 25 days before ripening) and S6 (harvested at maturity) stages, and 12 cDNA libraries (with three independent biological replicates for each sample) were constructed. In total, 37.85–43.05 million clean reads were obtained (Table S1). The mapping data revealed that 92.60 to 94.30% of clean reads were successfully mapped to the ‘Sanyueli’ plum reference genome. We obtained approximately 74.28 GB of clean reads, with Q30 ≥ 92.66% and GC content ranging from 45.41% to 46.22%. The data was considered to be reliable for further analysis of DEGs.

We normalized the expression level of each gene to the FPKM value to further explore the gene expression differences between FS and FY fruits. The Pearson correlation coefficients of the biological replicates for each sample ranged from 0.88 to 0.981 (Figure S1). We performed PCA analysis of gene expression values (FPKM) for all samples (Figure S2). The PCA score plots showed that the four inter-group samples were scattered, and the S5_FS samples were the most distant from other groups, while the intra-group samples were clustered together, indicating significant inter-group variation and good reproducibility of the intra-group samples. These results verified the quality of the obtained reads.

**Figure 5:**
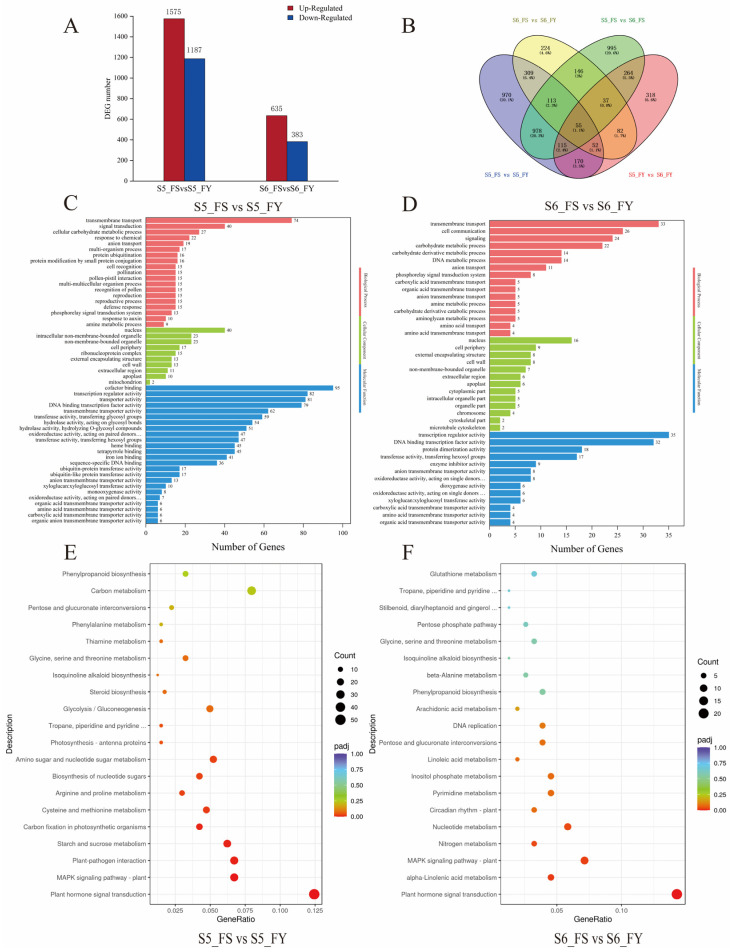
Differentially expressed genes (DEGs) in ‘Fengtangli’ plum from two pollination combinations. (**A**) Numbers of up-regulated and down-regulated DEGs of S5_FS vs. S5_FY and S6_FS vs. S6_FY. (**B**) Venn diagram of DEGs of S5_FS vs. S5_FY, S6_FS vs. S6_FY, S5_FS vs. S6_FS, and S5_FY vs. S6_FY. (**C**,**D**) GO enrichment analysis of DEGs of S5_FS vs. S5_FY and S6_FS vs. S6_FY. (**E**,**F**) KEGG pathway enrichment analysis of DEGs of S5_FS vs. S5_FY and S6_FS vs. S6_FY.

Differentially expressed genes (DEGs) were identified for |log2 foldchange| ≥1 and *p*-value ≤ 0.05 and comparisons were conducted between the FS and FY groups. Overall, 2762 DEGs were identified between FS and FY fruits at S5 stage, among which, compared with FY (S5), 1575 DEGs were up-regulated and 1187 DEGs were down-regulated in FS (Figure 5A). At S6 stage, the number of DEGs decreased to 1018 between FS and FY. Among them, 62% of DEGs were up-regulated in FS. The results indicated an obvious difference in metaxenia effect on fruit transcript levels at the ripening stage. Correspondingly, the Venn diagram illustrated the number of DEGs in different samples and stages (Figure 5B). There were 529 DEGs shared by S5_FS vs. S5_FY and S6_FS vs. S6_FY. 471 DEGs were also shared in S5_FS vs. S6_FS and S5_FY vs. S6_FY as the fruit developed. In addition, all comparison groups shared 392 DEGs.

### 2.7. Effects of Metaxenia on Functional Enrichments of DEGs

The Gene Ontology (GO) analysis was performed to classify the roles of DEGs in different groups, including biological process, cellular component, and molecular function (Figure 5C,D; Tables S2 and S3). In the S5_FS vs. S5_FY and S6_FS vs. S6_FY comparison pairs, 712 and 550 GO terms were enriched, respectively. GO terms related to biological process at S5 stage included pollination (GO:0009856), pollen-pistil interaction (GO:0009875), recognition of pollen (GO:0048544), reproduction (GO:0000003), reproductive process (GO:0022414), and response to auxin (GO:0009733). In the GO category of cellular component, cell periphery (GO:0071944), cell wall (GO:0005618) were mainly enriched. In terms of molecular function, DEGs were mainly associated with binding and catalytic activity, such as cofactor binding (GO:0048037), DNA binding transcription factor activity (GO:0003700), and oxidoreductase activity (GO:0016705). The results showed that biological metabolisms were changed in FS and FY due to the metaxenia effect.

We mapped DEGs to the reference pathways in the Kyoto Encyclopedia of Genes and Genomes (KEGG) database (Figure 5E,F; Tables S4 and S5). In FS vs. FY comparisons at both S5 and S6 stages, plant hormone signal transduction was the most significantly enriched pathway, and had the highest number of DEGs, 50 and 22, respectively. Among them, 72% and 82% of DEGs expression was upregulated in FS, respectively. Plant-pathogen interaction, starch and sucrose metabolism, carbon fixation in photosynthetic organisms, cysteine and methionine metabolism were significantly enriched in S5_FS vs. S5_FY comparison. Alpha-linolenic acid metabolism and nitrogen metabolism were significantly enriched in S6_FS vs. S6_FY comparison. Furthermore, MAPK signaling pathway was significantly enriched in both the S5_FS vs. S5_FY and S6_FS vs. S6_FY comparisons.

### 2.8. DEGs Related to Plant Hormone Signal Transduction

**Figure 6:**
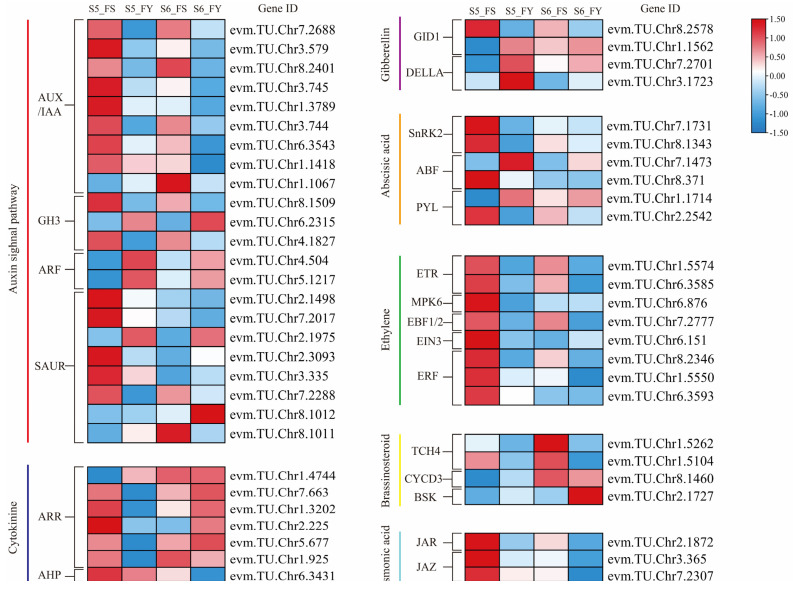
The expression profiles of DEGs in the plant hormone signal transduction pathways. Log_2_FPKM values were used for the heatmap analysis.

The hormone signal transduction pathway was the most significantly enriched KEGG pathway in the comparison of FS and FY fruits. Plant hormones play an important role in the development and ripening of plum. We analyzed the expression pattern of 54 DEGs related to plant hormone (Figure 6; Table S6). The results showed that these DEGs involved in metaxenia response, included auxin, gibberellin, cytokinin, abscisic acid, ethylene, brassinosteroid, and jasmonic acid.

The highest number of DEGs were involved in auxin; 22 in total. The transcription of most auxin-related DEGs were up-regulated in FS fruits, including auxin-responsive protein nine *AUX/IAA* genes (evm.TU.Chr7.2688, evm.TU.Chr3.579, evm.TU.Chr8.2401, evm.TU.Chr3.745, evm.TU.Chr1.3789, evm.TU.Chr3.744, evm.TU.Chr71.1418 and evm.TU.Chr1.1067) and five *SAUR* genes (evm.TU.Chr2.1498, evm.TU.Chr7.2017, evm.TU.Chr2.3093, evm.TU.Chr3.335 and evm.TU.Chr7.2288), 2 IAA-amino acid synthetase *GH3* genes (evm.TU.Chr8.1509 and evm.TU.Chr4.1827). However, the expression levels of auxin response factors *ARF* (evm.TU.Chr4.504 and evm.TU.Chr5.1217) were higher in FY than in FS fruits.

Seven DEGs related to cytokinin signaling were identified. In addition to evm.TU.Chr1.4744, the remaining five response regulator *ARR* genes (evm.TU.Chr7.663, evm.TU.Chr1.3202, evm.TU.Chr2.225, evm.TU.Chr5.677, and evm.TU.Chr1.925) and one histidine phosphotransfer protein *AHP* gene (evm.TU.Chr6.3431) were up-regulated in the S5_FS group. With fruit ripening, these DEGs showed opposite expression patterns. The expression levels of the DEGs in FY were significantly higher than in FS at the S6 stage. Furthermore, the expression of one gibberellin receptor *GID1* (evm.TU.Chr1.1562) and two *DELLA* proteins (evm.TU.Chr7.2701, evm.TU.Chr3.1723) were down-regulated in FS fruits at S5 and S6 stages.

Abscisic acid and ethylene play a positive role in regulating fruit ripening. Six and eight ABA- and ethylene-related DEGs were identified, respectively. Most of them showed up-regulated expression in FS fruits at S5 and S6 stages, including ABA receptor *Sucrose non-fermenting 1-related protein kinases 2* (*SnRK2*) (evm.TU.Chr7.1731, evm.TU.Chr8.1343) and *PYR1-like protein* (*PYL*) (evm.TU.Chr2.2542), ABRE binding factor *ABF* (evm.TU.Chr8.371), ethylene receptor *ETR* (evm.TU.Chr1.5574 and evm.TU.Chr6.3585), EIN3-binding F-box protein *EBF1/2* (evm.TU.Chr7.2777), ethylene insensitive 3 protein *EIN3* (evm.TU.Chr6.151), and ethylene-responsive transcription factor *ERF* (evm.TU.Chr8.2346, evm.TU.Chr1.5550 and 6.3593). 

Several other DEGs related to brassinosteroid and jasmonic acid also showed consistent expression patterns. The expression of xyloglucosyl transferase *TCH4* (evm.TU.Chr1.5262 and evm.TU.Chr1.5104), cyclin D-type protein *CYCD3* (evm.TU.Chr8.1460), BR-signaling kinase (evm.TU.Chr2.1727), jasmonic acid-amino synthetase *JAR* (evm.TU.Chr2.1872), jasmonate ZIM domain-containing protein *JAZ* (evm.TU.Chr3.365 and evm.TU.Chr7.2307) were up-regulated in FS fruits.

Overall, the expression levels of many DEGs related to plant hormone signaling pathways were down-regulated with fruit ripening. The expression of most DEGs was significantly higher in ‘Siyueli’-pollinated fruits than in ‘Yinhongli’-pollinated fruits.

### 2.9. DEGs Related to Starch and Sucrose Metabolism

**Figure 7:**
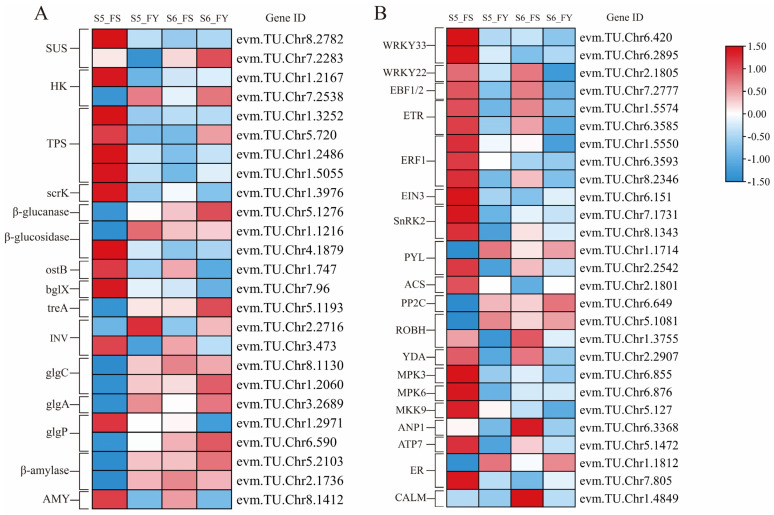
The expression profiles of DEGs enriched in the (**A**) starch and sucrose metabolic pathways and (**B**) MAPK signaling pathway. Log_2_FPKM values were used for the heatmap analysis.

In the starch and sucrose metabolic pathways, a total of 25 DEGs were identified (Figure 7A; Table S7). Pollination of ‘Siyueli’ pollen significantly up-regulated the expression levels of sucrose synthase (*SUS*) (evm.TU.Chr8.2782), trehalose-6-phosphate synthase (*TPS*) (evm.TU.Chr1.3252), fructokinase (*ScrK*) (evm.TU.Chr1.3976), trehalose 6-phosphate phosphatase (*ostB*) (evm.TU.Chr1.747), beta-glucosidase (*bglX*) (evm.TU.Chr7.96), beta-fructofuranosidase (INV) (evm.TU.Chr3.473), glycogen phosphorylase (*glgP*) (evm.TU.Chr1.2971), and alpha-amylase (*AMY*) (evm.TU.Chr8.1412); there were opposite expression profiles in FY fruits. In contrast, pollination of ‘Yinhongli’ pollen up-regulated the expression level of β-glucanase (evm.TU.Chr5.1276), β-glucosidase (evm.TU.Chr1.1216), alpha, alpha-trehalase (*treA*) (evm.TU.Chr5.1193), glucose-1-phosphate adenylyltransferase (*glgC*) (evm.TU.Chr8.1130, evm.TU.Chr1.2060), starch synthase (*glgA*) (evm.TU.Chr3.2689), and β-amylase (evm.TU.Chr5.2103, evm.TU.Chr2.1736). The expression levels of these genes were down-regulated in FS fruits.

### 2.10. DEGs Related to MAPK Signaling Pathway

In addition to the involvement of DEGs in the above pathways, pollen sources also had an impact on the regulation of MAPK signaling pathway. A total of 27 DEGs were identified in the FS vs. FY comparisons (Figure 7B; Table S8). Among them, 85% (23/27) of the DEGs were up-regulated in FS fruits compared with FY fruits at S5 stage, while 100% (11/11) of the DEGs were up-regulated in FS fruits at S6 stage. These up-regulated genes mainly include *EBF* and *ETR* (ethylene-responsive transcription factor), *EIN3* (ethylene-insensitive protein 3), *ACS* (ethylene synthase), *SnRK2* and *PYL2* (abscisic acid receptor), *WRKY 33* and *WRKY 22* (*WRKY* transcription factor), *MPK3*, *MPK6*, *MKK9*, *ANP1* (mitogen-activated protein kinase), *ATP7* (copper-transporting ATPase). In contrast, *PP2C* (protein phosphatase 2C), *ROBH* (respiratory burst oxidase), and *ER* (LRR receptor-like serine) were more highly expressed in FY than in FS.

### 2.11. qRT-PCR Verification for RNA-seq Data

**Figure 8:**
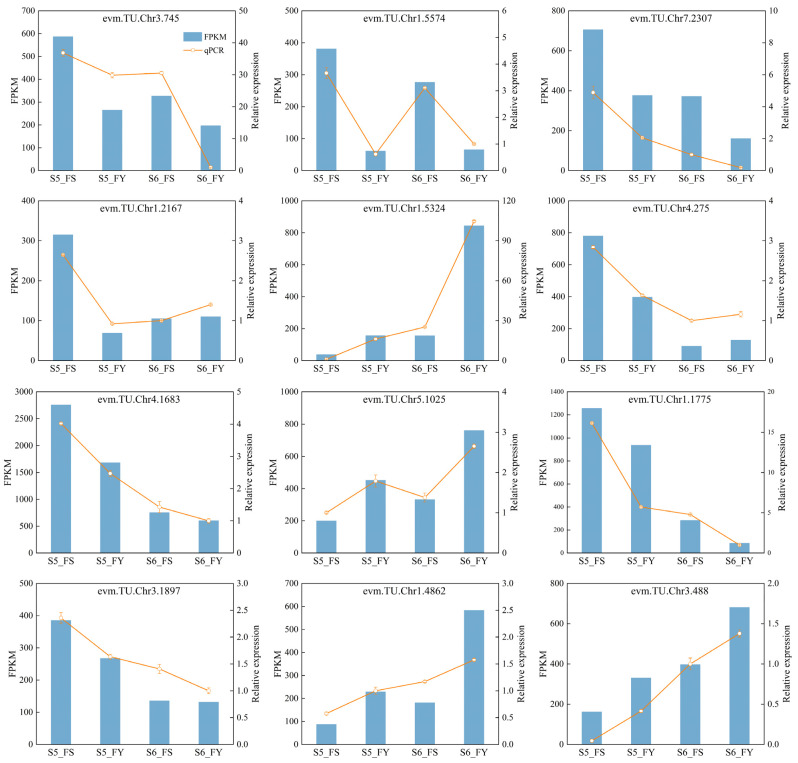
Real-time quantitative PCR validation profiles of twelve randomly selected genes from FS and FY fruits. The vertical bar indicates the standard error. The Y-axis on the left is the gene FPKM from RNA-seq (blue histogram). The y-axis on the right indicates the gene relative expression levels from the corresponding qRT-PCR analysis (orange line).

In order to verify the accuracy and authenticity of the transcriptome data, 12 genes were randomly selected for qRT-PCR analysis (Figure 8). The result showed that the expression trends of these genes were consistent with the RNA-seq data, indicating that the transcriptome data and our analysis are reliable.

## 3. Discussion

**Figure 9:**
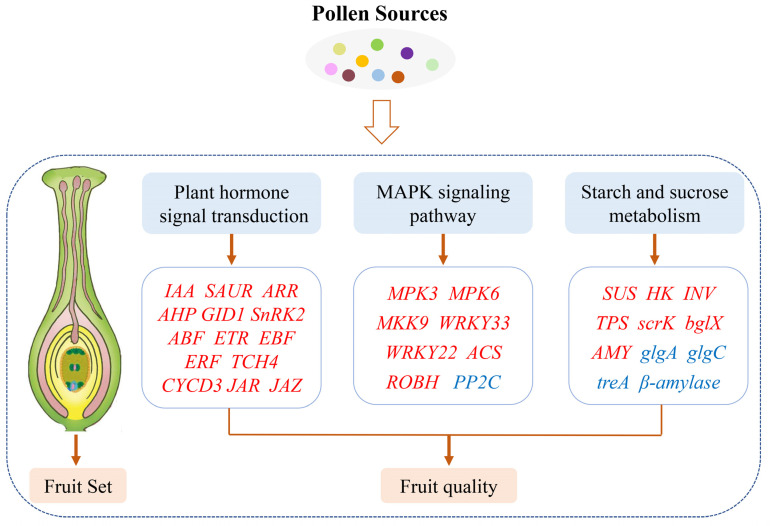
Physiological and molecular mechanisms by which different pollen sources affect fruit set and fruit quality in ‘Fengtangli’ plum. Red and blue colors indicate up- and down-regulation of gene expression in ‘Siyueli’-pollinated fruits, respectively.

Pollen tube growth and fruit set in *Prunus* species have been studied previously. However, there are few reports on Japanese plums. In the present study, pollen tube growth, fruit set rates, fruit characteristics and metaxenia effect of ‘Fengtangli’ plum with different pollination combinations were investigated for the first time. Our results suggested that pollen sources affecting fruit set rate by affecting the percentage of pistils with pollen tubes penetrating the ovary. The transcriptome analysis revealed that plant hormone signal transduction, starch and sucrose metabolism, and MAPK signaling pathway contributed to variations in fruit quality (Figure 9). We hypothesize that diverse pollen cause differences in fruit characteristics of ‘Fengtangli’ from different pollination combinations by regulating the expression of genes related to plant hormone and sugar metabolism during fruit development.

### 3.1. Pollen Germinability, Pollen Tube Growth and Fruit Set

The pollination process in fruit trees is the transfer of pollen grains from the stamens to the stigma [37]. Pollen viability is a critical factor for fertilization success and can generally be characterized by pollen germination rate in vitro. Pollen germinability is related to genetic characteristics, tree nutrition, climate, and management practices [38,39]. Previous research defined that the threshold between poor and good pollen germination in plums is 25% [40]. In this study, pollen germinability of all four cultivars was good, ranging from 34.47% to 51.22% (Figure 1A), suggesting that they could meet the pollination requirements. The result was consistent with Roberto et al. [41], who reported that the maximum pollen germination of several Rosaceae species including plum, almond, and peach, ranged from 30% to 52%. A higher pollen germinability may increase the probability of stigma receiving vigorous pollen and improve the effectiveness of pollination. The highest pollen germinability was recorded from ‘Fenghuangli’, followed by ‘Siyueli’, ‘Yinhongli’, while the lowest grain germination was recorded from ‘Fengtangli’. The same trend was observed in fruit set rates of the pollination combinations with them as pollen donors (Figure 1).

Furthermore, pollen tube elongation and penetration in the pistil are essential for successful fertilization [42,43]. All combinations in the present study showed a gradual decrease in the number of pollen tubes from the stigma to the base of the style and ovary (Figure 2), which is in accordance with Glisis et al. [40]. On the one hand, the pollen tube behavior may be attributed to GSI. Unlike sporophytic self-incompatibility, in which the inhibition occurs on the stigma, the pollen tube is arrested in the style in GSI [44]. On the other hand, it may be influenced by the nutrients in the transmitting tissue, such as glycoproteins, which have been confirmed to be a source of nutrients for pollen tube growth [45,46]. Additionally, plant metabolites such as flavonols and anthocyanins can affect pollen tube growth [47].

Cross-compatibility can be evaluated by the number of pollen tubes in the style and the final fruit set, while fruit set is significantly correlated to pollen tube growth (Figure 4A). The ‘Fengtangli’ × ‘Fenghuangli’ combination had the most pistils with pollen tubes at the base of the style and ovary, as well as the highest fruit set rate (Table 1, Figure 2B), implying that ‘Fenghuangli’ pollen was the most effective for inducing fruit set. ‘Siyueli’ and mixed pollen were the second-best, followed by ‘Yinhongli’ pollen. All three pollens were found to be compatible with ‘Fengtangli’. Future genetic identification of the *S*-locus can be used to further confirm the result. Partial compatibility occurs when the parents share one *S*-allele [44].

In the present study, pollen tube growth in the self-pollinated flowers was severely hindered from the upper to the base of the style, which is consistent with Nikolic and Milatovic [48], who claimed that the incompatibility of plum (*Prunus domestica* L.) occurs most often in the upper third of the style. Furthermore, abnormal morphology was often observed in incompatible pollen tubes, such as callose accumulation, apical branching, and tortuosity [40,49]. Irregular deposition of callose was noticed in the self-pollinated pollen tubes. However, 33% of pistils with pollen tubes at the base of the style were recorded in self-pollinated flowers. Guerra et al. [17] reported that cultivars were considered self-compatible when more than 40% of the pistils had at least one pollen tube reaching the base of the style. According to Kwon et al. [50] and Xu et al. [51], a fruit set rate above 5% in pollination combinations is considered compatible. ‘Fengtangli’ plum may be partially self-compatible due to a self-pollination fruit set rate of 12.20%, which lays the foundation for future research on ‘Fengtangli’ plum. Following this criterion, ‘Siyueli’, ‘Fenghuangli’, and ‘Yinhongli’ are all compatible with ‘Fengtangli’.

### 3.2. Metaxenia Effect on ‘Fengtangli’ Plum Fruit Characteristics

Since 1930, there have been many reports on metaxenia [52]. The effect of pollen sources on fruit characteristics has been observed in many species, such as citrus [19], hazelnut [53], raspberry [54]. In this study, diverse pollen sources influenced fruit size of ‘Fengtangli’ (Figure 3). The largest and heaviest fruits were obtained from flowers pollinated by ‘Siyueli’ and ‘Fenghuangli’. The smaller seeds and pulp thickness were determined in self-pollinated and open-pollinated fruits. Similar results were noticed by Zhang et al. [55], who found that different pollen sources affected fruit set rate, fruit size, and weight of ‘Frinar’ plum. These can be attributed to the metaxenia effect, caused by variations in endogenous hormone levels in the fruit [25]. By transcriptome sequencing, we found that plant hormone signal transduction pathways were significantly enriched in ‘Fengtangli’ fruits (FS and FY) pollinated by different pollen sources (‘Siyueli’ and ‘Yinhongli’ pollen). Auxin-related *IAA* genes, abscisic acid-related *SnRK2*, ethylene-related *ETR*, *EBF1/2*, *ERF*, brassinosteroid-related *TCH4*, *CYCD3*, and jasmonic acid-related *JAR* and *JAZ* genes were expressed up-regulated in FS fruits. The gibberellin-related *GIDI* and *DELLA* genes, however, were up-regulated in FY fruits. All these hormones are associated with fruit growth, development, and ripening. Hormones such as auxin and gibberellin, which modulate fruit formation and development by regulating cell division and expansion, are produced in response to pollination and fertilization [56]. ‘Siyueli’ pollen commonly induced up-regulation of the expression of these genes, suggesting that pollen may regulate fruit traits by affecting the expression of phytohormone-related genes. In addition, the MAPK signaling system plays an important role in physiological processes such as plant growth and development, disease resistance and other responses to stress [57]. In the present study, the expression of MPK3/MPK6 and its downstream *WRKY33* and *WRKY22* expression were up-regulated in ‘Siyueli’-pollinated fruits. We suggest that the plant hormone pathway and the *MPK3/MPK6-WRKY33* module act synergistically at multiple levels to regulate fruit development, resulting in differences in FS and FY fruit traits. The expression levels of both ABA receptors *SnRK2* and *PYL* and ethylene receptors *EBF1/2*, *ETR*, *ERF* and *EIN3* in the MAPK signaling pathway are up-regulated in FS. This synergistic effect has also been reported in Arabidopsis [58].

Pollen sources affected the internal quality, including soluble solids and sugar content. It may be due to differences in the activity of key enzymes [59]. The soluble sugar content in FS fruits was significantly higher than that in FY fruits. This was associated with DEGs involved in starch and sucrose metabolism in plum fruits. On the one hand, the expression of *SUS* (encoding sucrose synthase) was up-regulated in FS fruits. The up-regulated expression of *INV* genes mediated the irreversible reaction catalyzing the conversion of sucrose to glucose and fructose [60]. β-glucosidase (*bglX*) was up-regulated to promote glucose accumulation. On the other hand, *glgC* and *glgA* genes involved in starch synthesis were down-regulated, while the *AMY* gene related to starch degradation was up-regulated in expression, potentially facilitating the breakdown of starch to glucose [61]. However, the expression of β-amylase and *treA* (associated with starch degradation) was up-regulated in FY fruits. This is similar to Li et al. [26] who reported significant differences in starch and sugar contents of chestnuts from various pollination combinations, which were associated with DEGs encoding key enzymes in the starch and sugar metabolic pathways.

Titratable acid, however, did not differ among treatments. Similar findings have been documented in apple and ‘Clementine’ citrus [19,29]. There is little difference in firmness between pollination combinations (ranging from 30.61 N to 35.84 N), which is greatly affected by the picking date. In addition, significant positive correlations were discovered between fruit size and soluble solids and sugar content, suggesting that larger and heavier fruits may tend to accumulate more sugar. High firmness, soluble solids, and soluble sugar content from the ‘Fengtangli’ × ‘Siyueli’ combination satisfy consumer demand for crispy, sweet plum fruits. The second best was obtained from ‘Fenghuangli’-pollinated fruits, while self-pollinated and open-pollinated fruits possessed low levels for most of the evaluated characteristics. The pollen source of fruit obtained from open pollination was not clear, which may contribute to lower mean fruit quality than ‘Siyueli’ pollination and ‘Fenghuangli’ pollination. In this work, the effect of mixed pollen pollination on plum fruit largely reflected the performance of the female parent. Therefore, comparing the effect of different pollen sources with mixed pollen on plum fruit can reflect the metaxenia effect.

## 4. Materials and Methods

### 4.1. Plant Materials

Four commercial Japanese plum cultivars were used in this study: ‘Fengtangli’, ‘Siyueli’, ‘Fenghuangli’, and ‘Yinhongli’. They were grown in an orchard situated in Suining City, Sichuan Province, China (altitude 450 m; latitude 31°10′ N; longitude 105°3′ E). The average long-term annual temperature in that location is about 17 °C and the precipitation is about 927 mm. All the cultivars were maintained with the same water-fertilizer schedule and management.

### 4.2. Pollen Germinability

For preparing pollen samples, flowers of ‘Fengtangli’, ‘Siyueli’, ‘Fenghuangli’, and ‘Yinhongli’ were collected at the balloon stage. The anthers were removed and placed on paper, and subsequently dried in the oven at 25 °C for 24 h. Then pollen grains were extracted and stored at −20 °C for pollination.

Pollen grains were dispersed on a solidified germination medium made up of 1% agar, 10% sucrose, and 0.03% H_3_BO_3_ [41]. After incubation at 25 °C for 4 h, pollen germination was observed under a microscope (Olympus CX21, Tokyo, Japan). Pollen grains with tubes longer than their diameter were deemed to have germinated [40]. Pollen germinability was recorded in three petri dishes for each cultivar by counting germinated pollen grains in three microscopic fields per plate, each field containing at least 100 pollen grains.

### 4.3. Pollination Experiments

‘Fengtangli’ was used as a female parent (pollen recipient). ‘Siyueli’, ‘Fenghuangli’, and ‘Yinhongli’ were selected as pollen donors. We established the following pollination combinations: ‘Fengtangli’ × ‘Siyueli’ (FS), ‘Fengtangli’ × ‘Fenghuangli’ (FF), ‘Fengtangli’ × ‘Yinhongli’ (FY), mixed pollination (MP) with a pollen mix of the four plum cultivars, self-pollination (SP), and open pollination (OP).

Pollination procedure was performed as previously described by Glisic et al. [40] and Cerovic et al. [62]. Eighteen uniform and healthy five-year-old ‘Fengtangli’ plum trees were chosen, with three trees for each pollination combination. On each tree, at least four branches with sufficient flowers were selected and tagged. Flowers at the balloon stage were emasculated and others were removed. Then, the emasculated flowers were isolated in parchment bags (28 cm × 17 cm) to prevent uncontrolled pollination. Approximately 450–500 flowers were prepared for each pollination combination. ‘Fengtangli’ pistils were hand pollinated with prepared pollen samples using a small brush on the first day of full flowering, when ‘Fengtangli’ flowers showed high stigma receptivity. After pollination, all the pollinated flowers were bagged again for 4 weeks. Open-pollinated branches were marked only and were not hand-pollinated or bagged.

### 4.4. Evaluation of Fruit Set

The number of fruit sets was counted five weeks after pollination. The fruit set rate was calculated as the percentage of fruits to pollinated flowers.

### 4.5. Pollen Tube Growth In Vivo

The pollinated flowers were collected 96 h after pollination, fixed in FAA solution (40% formaldehyde: acetic acid: 70% ethanol, 5:5:90), and stored at 4 °C [19]. At least 20 pistils for microscopic examination were prepared according to Chen and Fang [63]. Pistils were softened and stained with 0.1% aniline blue in 0.1 N K_3_PO_4_ [64]. Pistils were observed under a fluorescence microscope (Olympus BX53, Tokyo, Japan) to determine the percentage of pollen germination on stigma, the mean number of pollen tubes, and the percentage of pistils with pollen tubes in the style (upper third and the base) and ovary.

### 4.6. Determination of Fruit Characteristics

‘Fengtangli’ fruit samples were harvested at maturity from branches marked by each pollination combination. Thirty fruits from each treatment were used for the determination of fruit characteristics. Single fruit weight was measured with an electronic balance; the length and diameter of fruit and seed, as well as pulp thickness, were measured with vernier calipers. Fruit shape index represents the ratio of fruit length to diameter. Firmness was determined with a fruit texture analyzer (TMS-Pro, FTC, Sterling, VA, USA). Total soluble solids (TSS) were measured with a digital refractometer and expressed as degrees of Brix (%). Titration with a sodium hydroxide indicator was used to determine the titratable acid (TA) content of the fruits. The anthrone-sulfuric acid method was used to determine the soluble sugar content (%). The sugar-acid ratio was calculated as the soluble sugar/TA ratio.

### 4.7. RNA Extraction, Library Construction and Sequencing

The plums (FS and FY, with significant differences in fruit quality) harvested 25 days before ripening (S5) and at maturity (S6) were used for RNA extraction, containing three biological replicates for each experiment. RNA integrity was assessed using the RNA Nano 6000 Assay Kit of the Bioanalyzer 2100 system (Agilent Technologies, Santa Clara, CA, USA). The cDNA library construction and high-throughput sequencing were commissioned by Novogene Co., Ltd. (Beijing, China). Total RNA was used as input material for the RNA sample preparations. mRNA was purified using poly-T oligo-attached magnetic beads and then fragmented. First strand cDNA was synthesized using random hexamer primer, followed by synthesis of second strand cDNA. cDNA fragments of preferentially 370~420 bp in length were selected. Sequencing libraries were generated via PCR. PCR products were purified (AMPure XP system)(Beckman Coulter, Fullerton, CA, USA) and library quality was assessed on the Agilent Bioanalyzer 2100 system. The library preparations were sequenced on an Illumina Novaseq platform and 150 bp paired-end reads were generated.

### 4.8. Analysis of the RNA-seq Data

Reads containing adapter and poly-N, and low-quality reads were removed from raw data to obtain clean data. Q20, Q30 and GC content of the clean data were calculated. All generated clean reads were mapped to ‘Sanyueli’ plum reference genome (https://www.rosaceae.org/Analysis/9450778, accessed on 12 July 2022) using Hisat2 v2.0.5 [65]. FeatureCounts v1.5.0-p3 [66] was used to count the reads numbers mapped to each gene. The expression level of genes was quantified by FPKM (number of fragments per kilobase of the transcript sequence per million base pairs sequenced).

Pearson’s correlation and PCA was performed to show the correlations between biological replicates. The differential expression analyses between FS and FY fruit samples were performed using the DESeq2 R package (1.20.0) [67]. Genes with |log2 foldchange| ≥ 1 and *p*-value ≤ 0.05 were considered as differentially expressed genes (DEGs) in comparative analysis.

### 4.9. Functional Classification and Pathway Enrichment

Gene Ontology (GO) enrichment analysis of DEGs was implemented by the clusterProfiler R package. GO terms with corrected *p*-value less than 0.05 were considered significantly enriched by DEGs. The Kyoto encyclopedia of genes and genomes (KEGG) pathway analysis was executed to retrieve the enriched pathways.

### 4.10. Quantitative Real-Time PCR (qRT-PCR) Assay

Total RNA was extracted from plum fresh (FS and FY) at S5 and S6 stages using the RNAprep Pure Plnat Kit (TIANGEN, Beijing, China). First-strand cDNA was synthesized using an RNA reverse transcription kit (Mei5bio, Beijing, China). We used Primer Premier 5 to design specific primers for 12 genes (Table S9). The qRT-PCR analysis was performed using a CFX96 system (Bio-Rad, Hercules, CA, USA) and SYBR Premix EsTaq (Mei5bio, Beijing, China) to validate RNA-seq data. All samples had three biological replicates and were technically replicated three times. The 2^−∆∆CT^ method were used to calculate the relative expression level of the selected DEGs [68]. The amplification of *Actin* (evm.TU.Chr6.1716) sequence was used as an endogenous reference to normalize all the data.

### 4.11. Statistical Analysis

The data were statistically analyzed by one-way analysis of variance (ANOVA) using SPSS 27.0 (IBM, Armonk, NY, USA), and Duncan’s Multiple Range test (DMRT) was used to check for significant (*p* < 0.05) treatment differences. Pearson’s correlation analysis (*p* < 0.05) was applied to assess the relationship between pollen tube growth parameters and fruit set, as well as fruit characteristics.

## 5. Conclusions

In fact, a single fruit tree species should be avoided in an orchard. Co-cultivation with suitable pollinizers is a good option for commercial production instead. In the present study, the pollen germinability of the four cultivars was good by microscopic observation, ranging from 34.47% to 51.22%. However, the pollination effects of diverse pollen sources differed significantly. The results of pollen tube growth and fruit set rate revealed that ‘Fenghuangli’ pollen is the best fruit set inducer. Moreover, metaxenia effects could be responsible for variations in fruit size, soluble solids, and soluble sugar content. ‘Siyueli’ pollen produced the best fruit quality. Transcriptome analysis revealed that ‘Siyueli’ pollen induced the up-regulation of expression of DEGs enriched in plant hormone signal transduction, starch and sucrose metabolism pathways. These findings enhance our understanding of pollen sources affecting fruit set and fruit characteristics and lay the foundation for the exploration of the mechanism of metaxenia effects.

## Figures and Tables

**Table 1:** Pollen germination on the stigma surface, percentage of pistils with pollen tubes in different parts of the style and ovary, number of pollen tubes in style.

Treatment	Pollen Grains Germination on Stigma (%)	Pistils (%) with Pollen Tubes			Number of Pollen Tubes		
		At the Upper Third of Style	At the Base of Style	Penetrated to Ovary	At the Upper Third of Style	At the Base of Style	Penetrated to Ovary
FS	61.64 ± 3.54 a ^1^	95.45 ± 4.55 a	54.55 ± 4.54 c	36.36 ± 3.76 ab	33.17 ± 5.49 b	11.33 ± 2.42 c	1.71 ± 0.49 b
FF	56.91 ± 5.66 ab	91.30 ± 4.35 a	78.26 ± 4.30 a	43.48 ± 2.22 a	47.33 ± 5.08 a	23.17 ± 4.31 a	2.57 ± 0.53 a
FY	53.20 ± 4.10 bc	92.31 ± 3.85 a	65.38 ± 4.01 b	26.92 ± 2.62 cd	26.50 ± 3.15 c	9.67 ± 5.09 cd	0.86 ± 0.38 cd
MP	63.72 ± 2.87 a	92.00 ± 4.00 a	76.00 ± 3.80 a	44.00 ± 2.40 a	31.83 ± 4.26 b	16.00 ± 4.15 b	1.53 ± 0.52 b
SP	48.14 ± 4.53 c	79.17 ± 4.17 b	33.33 ± 4.06 d	20.83 ± 3.39 d	21.00 ± 3.22 d	6.17 ± 1.47 d	0.43 ± 0.44 d
OP	45.92 ± 2.32 c	83.33 ± 4.16 b	67.67 ± 2.51 b	29.17 ± 4.17 bc	35.67 ± 4.32 b	19.00 ± 2.53 ab	1.29 ± 0.48 bc

^1^ The data are represented as mean ± standard deviation (SD). Means within a column followed by the same letter are not significant at *p* < 0.05.

## Data Availability

The raw reads from the Illumina Novaseq 6000 platform were deposited into NCBI in the sequence reads archive (SRA) under the BioProject accession number PRJNA888052.

## Supplementary Materials

The supporting information can be downloaded at: https://www.mdpi.com/article/10.3390/ijms232112959/s1.
