# Supplementary material for: Effects of Pollen Sources on Fruit Set and Fruit Characteristics of ‘Fengtangli’ Plum (Prunus salicina Lindl.) Based on Microscopic and Transcriptomic Analysis
Source: Int J Mol Sci. 2022 Oct 26;23(21):12959. doi: 10.3390/ijms232112959 (PMC9656660; doi:10.3390/ijms232112959)
Supplement: Supplementary file 1 [file ijms-23-12959-s001.zip › Supplementary Files/Supplementary Figures.pdf]

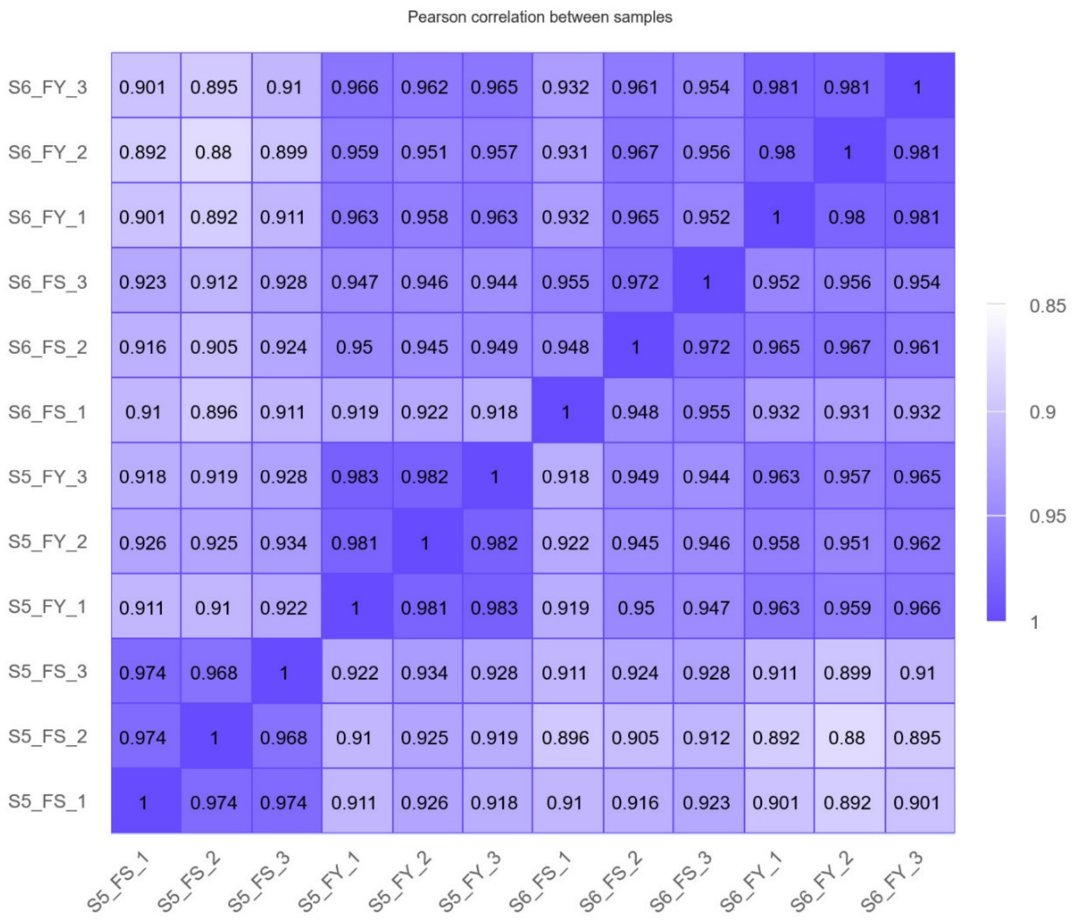

Figure S1. Pearson’s correlation matrix between different samples.

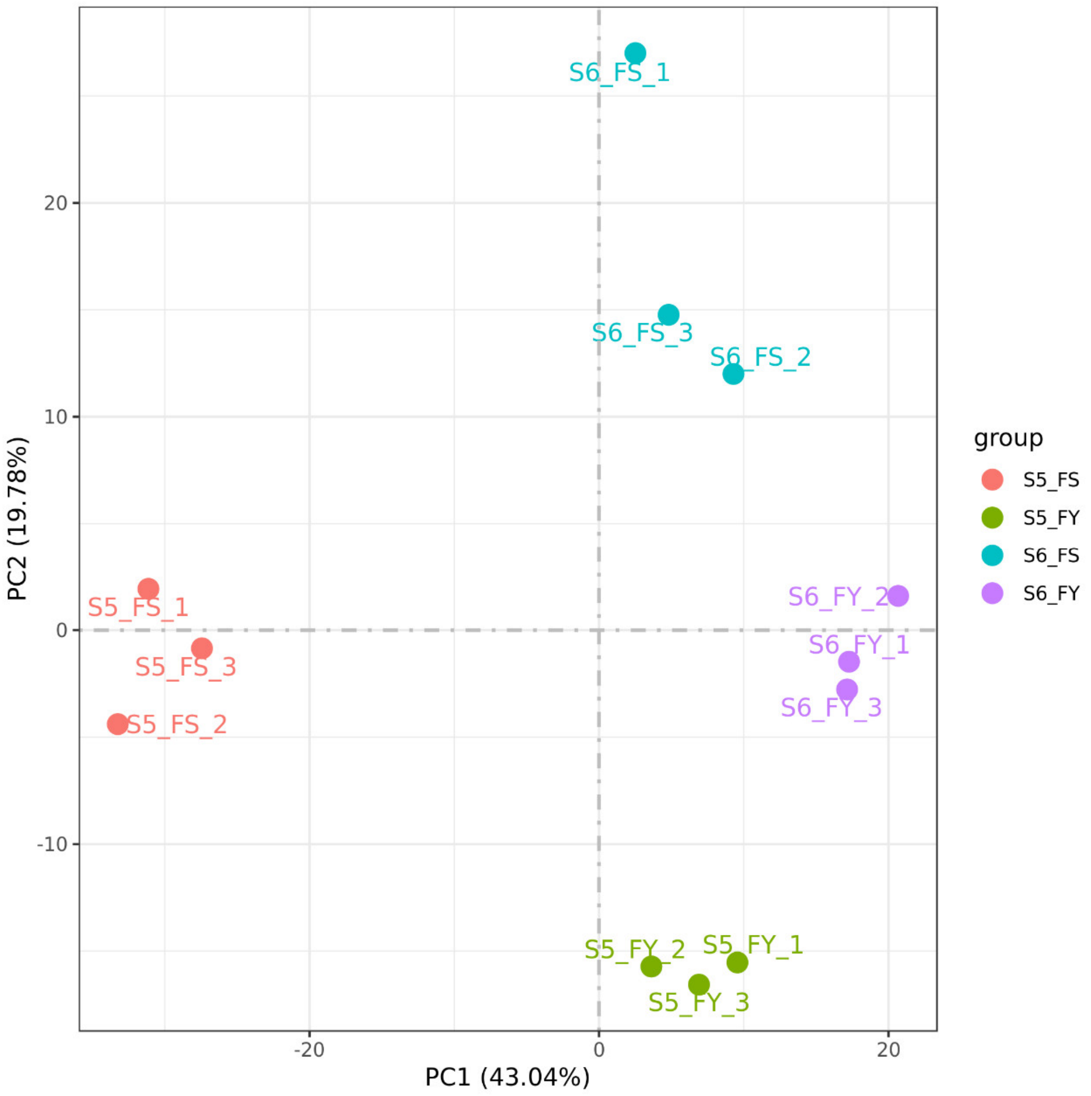

Figure S2. The result of principal component analysis (PCA) of all samples.
